# Benign duodenocolic fistula: A case report and review of the literature

**DOI:** 10.3389/fsurg.2022.1049666

**Published:** 2023-01-06

**Authors:** Zeyu Li, Wenjing Peng, Hongliang Yao

**Affiliations:** ^1^ Department of General Surgery, The Second Xiangya Hospital of Central South University, Changsha, China; ^2^ Department of General Medicine, The Second Xiangya Hospital of Central South University, Changsha, China

**Keywords:** duodenocolic fistula, duodenal ulcer, diarrhea, weight loss, barium meal

## Abstract

Duodenocolic fistula is a rare upper gastrointestinal fistula that can be benign or malignant. However, benign duodenocolic fistulas are particularly rare. Duodenocolic fistulas are often a complication of advanced colon cancer. The most common cause of benign fistulas is perforation of the duodenal ulcer. We report a case of a benign duodenocolic fistula in a patient who presented with abdominal pain, diarrhea, and weight loss. Gastroscopy and an upper gastrointestinal study confirmed the presence of the fistula. Surgery was performed, and the pathological examination demonstrated that the fistula originated from a duodenal ulcer.

## Introduction

Duodenocolic fistula is a rare type of upper gastrointestinal fistula. Perforated duodenal ulcers are usually the most common cause of benign fistulas (1). Malignant duodenocolic fistulas often arise from colon cancer in the proximal transverse colon or hepatic flexure (2). Clinical features include abdominal pain, diarrhea, and weight loss (3). The most remarkable symptom is diarrhea, which can occasionally contain food particles that have not yet been digested (4). A few patients present with fecaloid vomiting (5). Surgery is the primary treatment modality for this condition. We report a case of a benign duodenocolic fistula secondary to a duodenal ulcer. After surgery was performed, the symptoms of abdominal pain and diarrhea rapidly improved. The patient regained weight and recovered satisfactorily.

## Case report

A 53-year-old man presented with a 20-year history of abdominal pain, nausea, vomiting, and one episode of hematemesis. In April 2012, the abdominal pain was aggravated and was accompanied by edema of the lower extremities and abdominal bloating. Subsequent gastroduodenoscopy confirmed a duodenal ulcer. He was infected with Helicobacter pylori. The patient was treated with amoxicillin, clarithromycin, and protection of the gastric mucosa. Albumin infusion and diuretics were administered for hypoproteinemia and ascites. The patient's clinical condition had greatly improved and he was asymptomatic for 9 years. In November 2021, he developed abdominal pain and diarrhea and experienced the occasional passage of undigested food, general malaise, and slight edema of the lower limbs. He also experienced weight loss of 10 kg over the course of 6 months.

**Figure 1:**
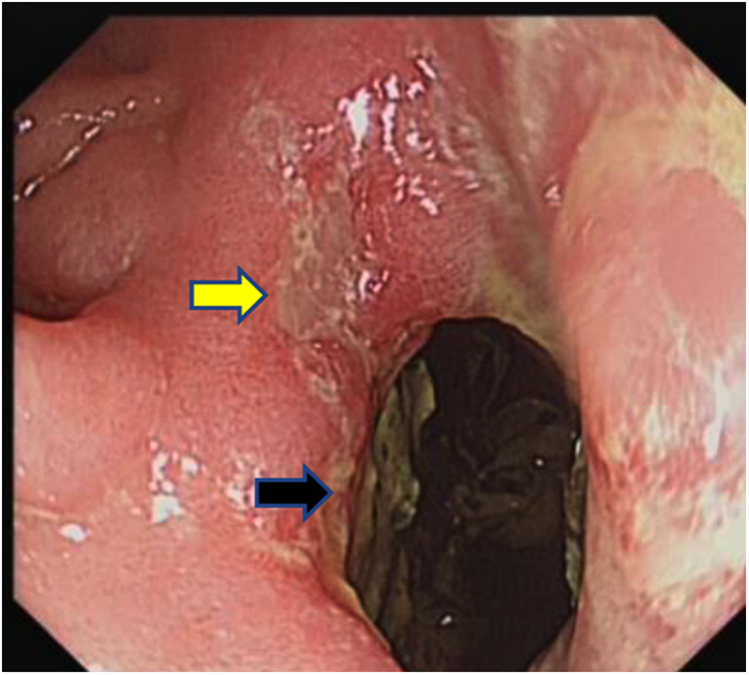
Gastroduodenoscopy showed a fistula with a diameter of 15 mm at the junction of the first and second parts of the duodenum, as well as an ulcer with a diameter of 8 mm next to the fistula (the black arrow indicates the fistula, and the yellow arrow indicates the ulcer). Fecal residue can be observed through the opening of the fistula.

**Figure 2:**
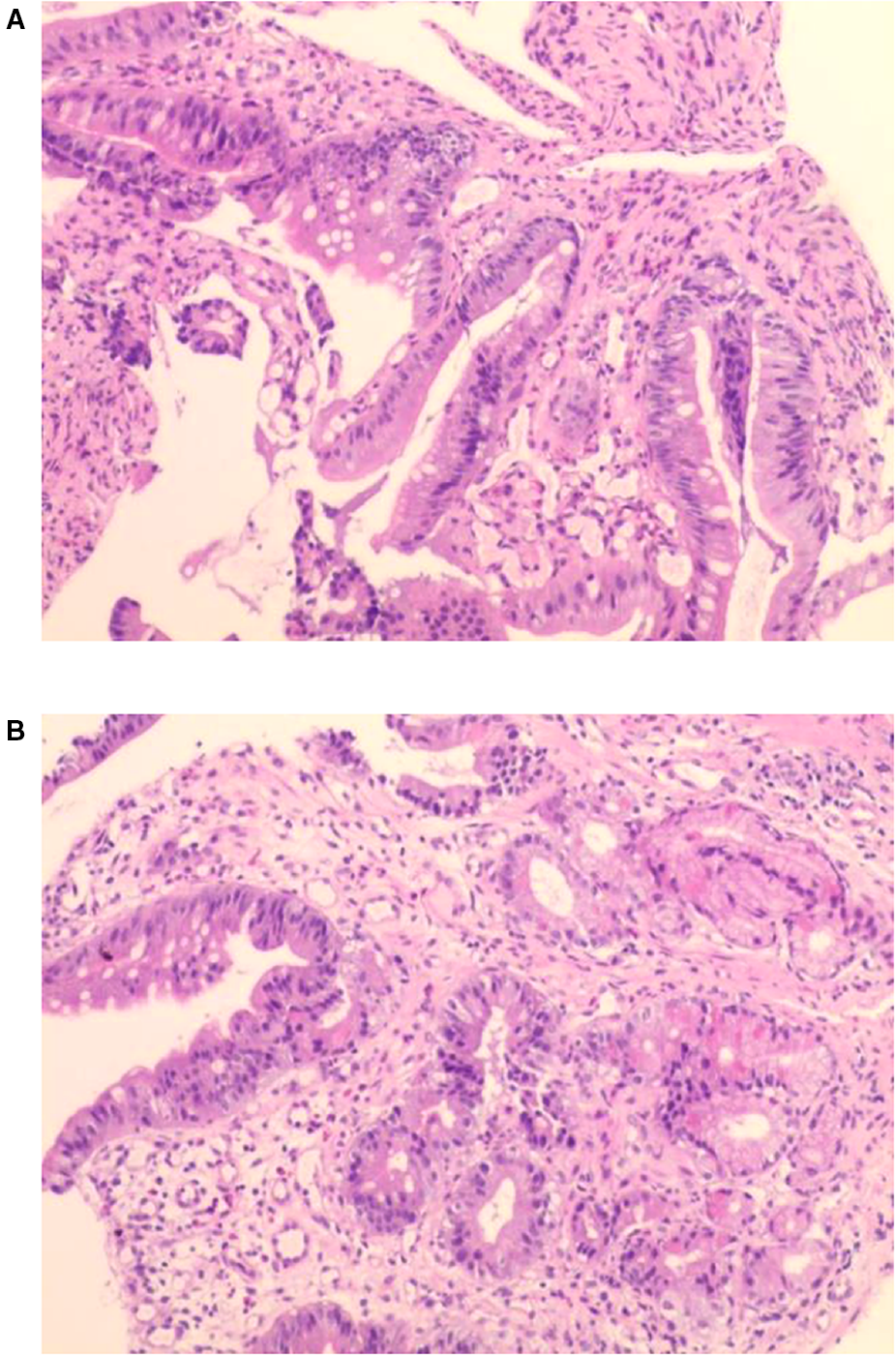
(**A,B**) Biopsy of the ulcer revealed chronic active inflammation of the mucosa and ulcer formation.

**Figure 3:**
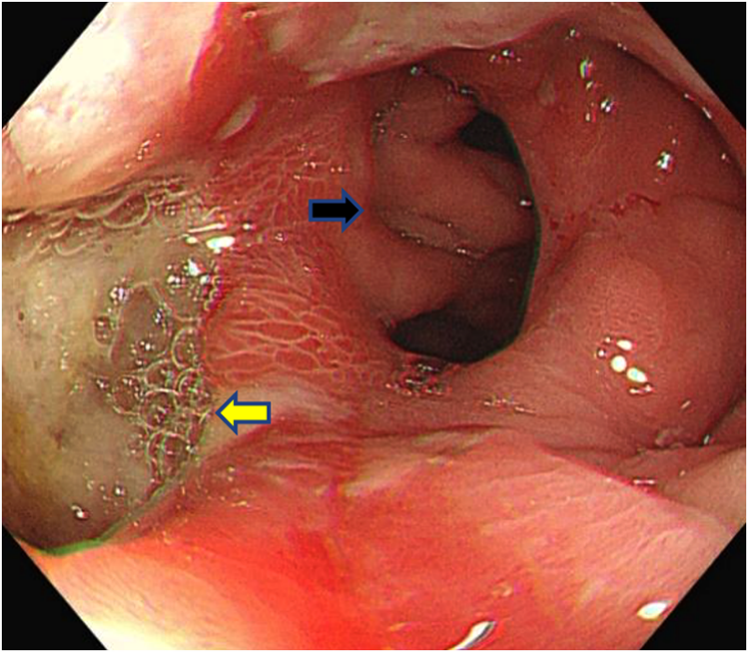
Colonoscopy confirmed the presence of the fistula and a giant ulcer can be seen in the hepatic flexure of the transverse colon (the black arrow indicates the fistula, and the yellow arrow indicates the ulcer).

**Figure 4:**
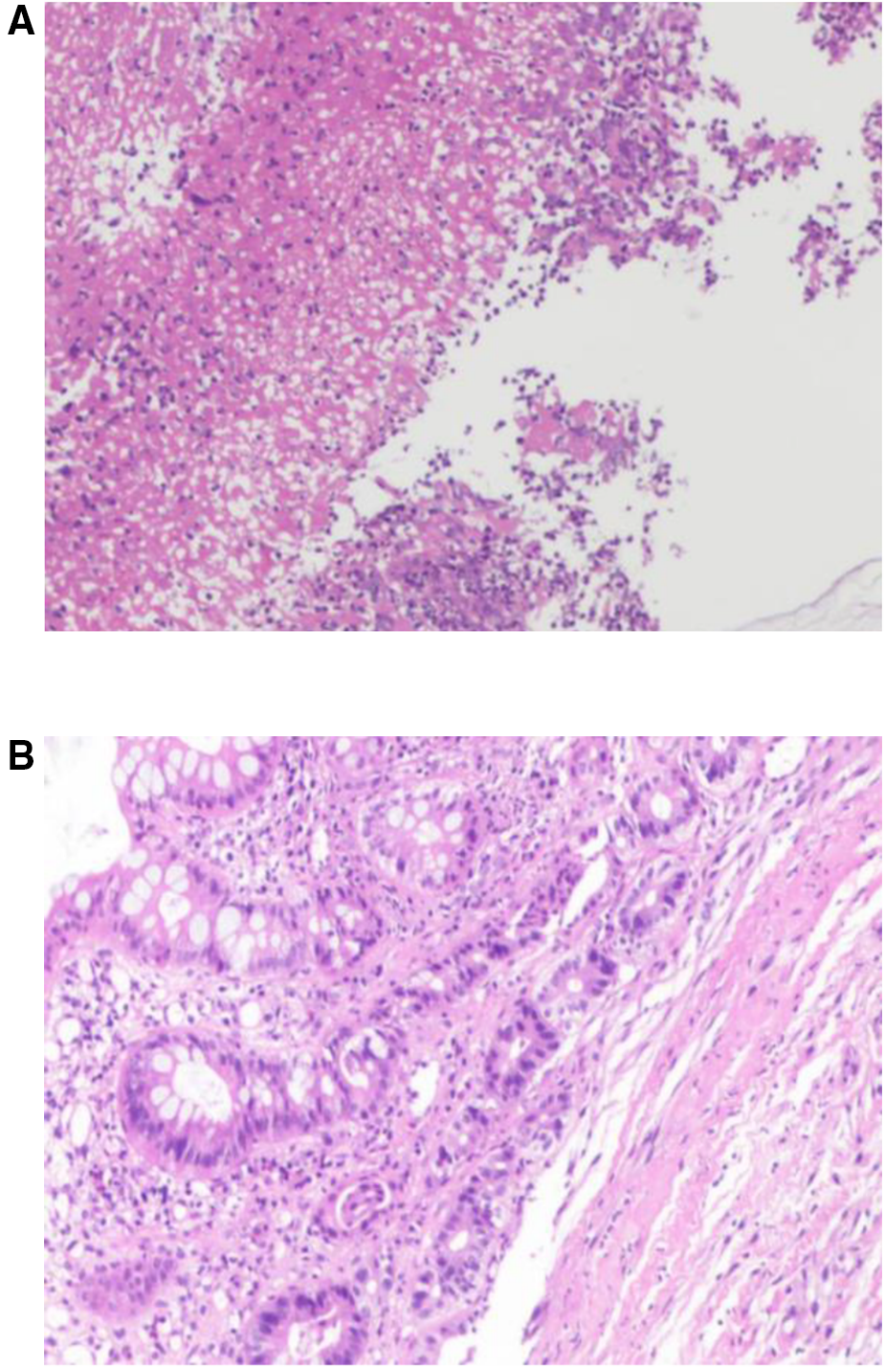
(**A,B**) Biopsy of the colonic ulcer showed chronic inflammation of the mucosa with ulcer formation.

**Figure 5:**
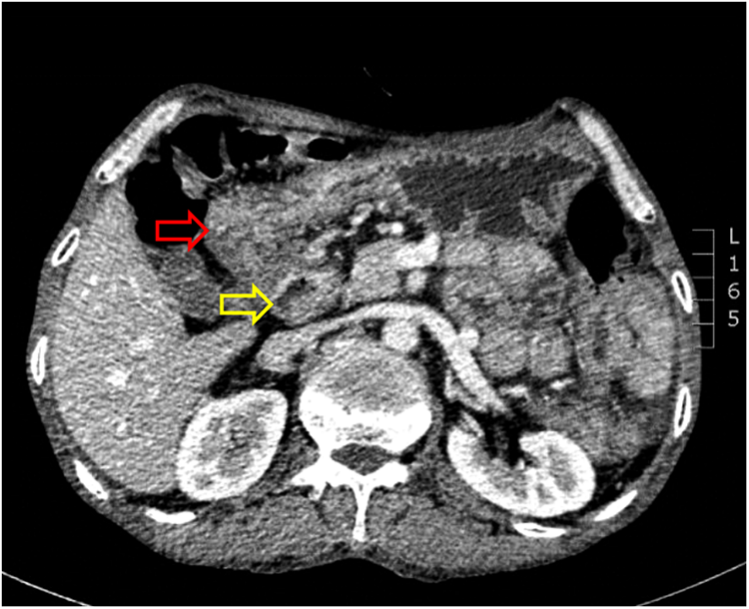
Computed tomography indicated no clear boundary between the descending duodenum and the transverse colon (the red arrow indicates the transverse colon, and the yellow arrow indicates the descending duodenum).

**Figure 6:**
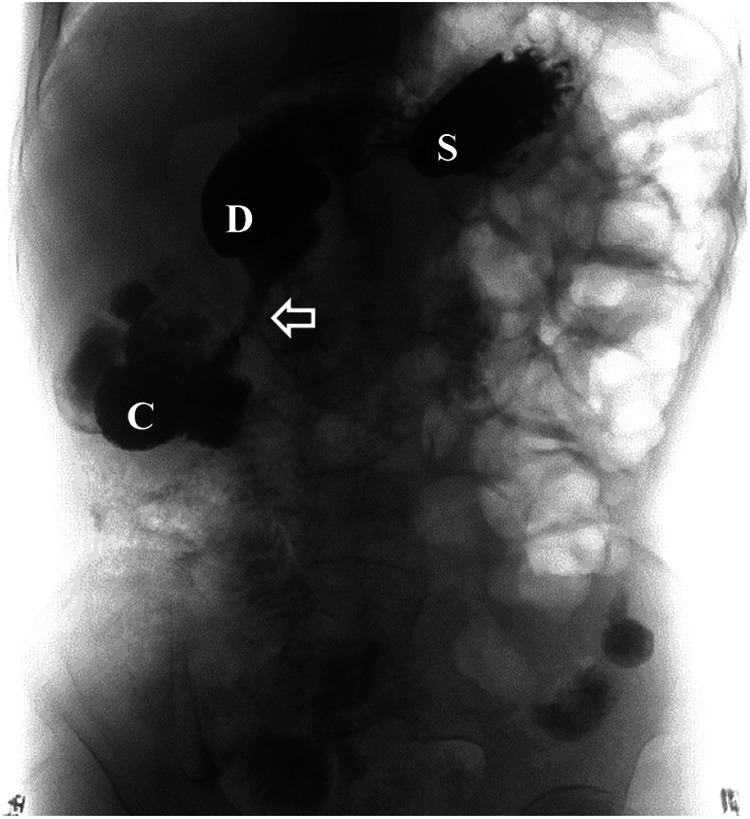
Upper gastrointestinal study revealed rapid transit of the contrast agent into the colon and confirmed the presence of a fistula (the white arrow indicates the fistula). C, colon; D, duodenum; S, stomach.

Examination revealed normal vital signs; however, the patient was thin and malnourished. His height was 165 cm and his weight was 49 kg. His body mass index was 17.99. The abdomen was soft, with no tenderness or palpable masses. His history was significant for duodenal ulcers for more than 10 years. Laboratory tests showed a serum albumin level of 26.9 g/L, hemoglobin level of 12.8 g/dl, potassium level of 2.7 mmol/L, and sodium level of 139 mmol/L. Gastroduodenoscopy revealed a fistula with a diameter of 15 mm at the junction of the first and second parts of the duodenum, as well as an ulcer with a diameter of 8 mm next to the fistula (Figure 1). A biopsy of the ulcer revealed chronic active inflammation of the mucosa and ulcer formation (Figures 2A,B). Colonoscopy confirmed the presence of a fistula, and a giant ulcer was observed in the hepatic flexure of the transverse colon (Figure 3). A biopsy of the colonic ulcer showed chronic inflammation of the mucosa with ulcer formation (Figures 4A,B). Computed tomography indicated no clear boundary between the descending duodenum and transverse colon (Figure 5). An upper gastrointestinal study revealed rapid transit of the contrast agent into the colon and confirmed the presence of a fistula (Figure 6).

After discussion at the multidisciplinary team meeting, we concluded that the fistula was most likely caused by a chronic penetrating duodenal ulcer. After a detailed discussion with the patient, we decided to perform surgery. Severe protein malnutrition and electrolyte imbalance were treated with intravenous hyperalimentation.

**Figure 7:**
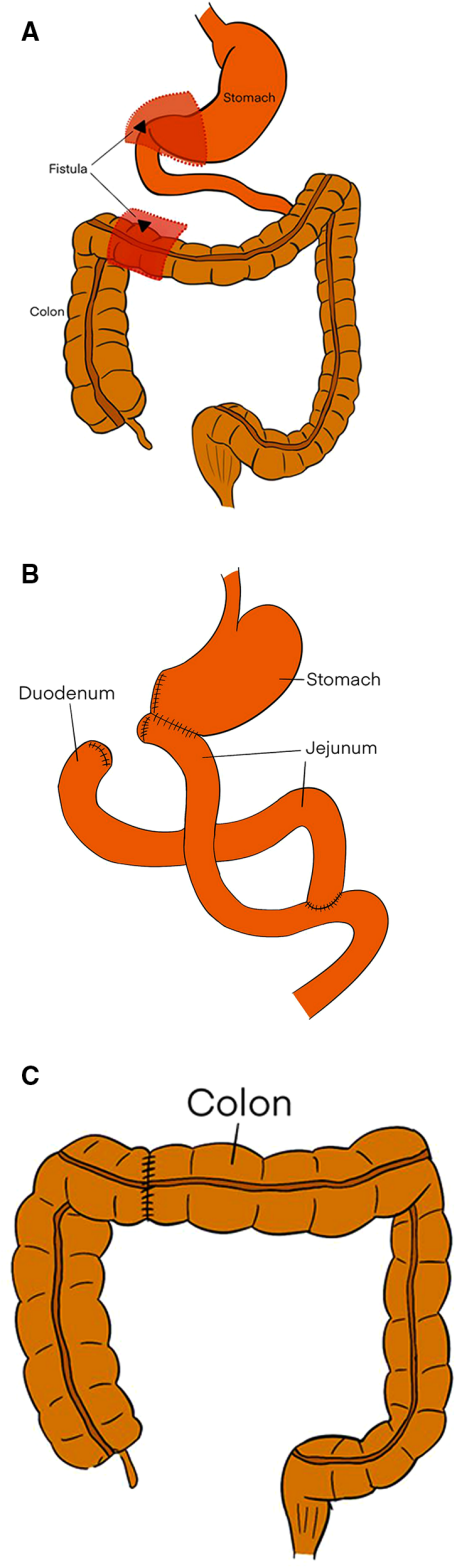
(**A**) The dotted lines and red regions indicate the resected the part of stomach, duodenum, and colon, including the fistula. (**B**) The Roux-en-Y gastric bypass. (**C**) The end-to-end intestinal anastomosis.

On March 17, 2022, surgical exploration indicated the presence of severe adhesions of the gastric antrum, duodenal bulb, and transverse colon, which were difficult to release. It is difficult to remove a fistula, and the fistula opening was located at the junction of the first and second portions of the duodenum. Accordingly, the patient underwent resection of the segmental duodenum, distal stomach, and partial transverse colon (Figure 7A). Roux-en-Y gastric bypass and end-to-end intestinal anastomosis were chosen to reconstruct the stomach and colon, respectively (Figure 7B,C).

**Figure 8:**
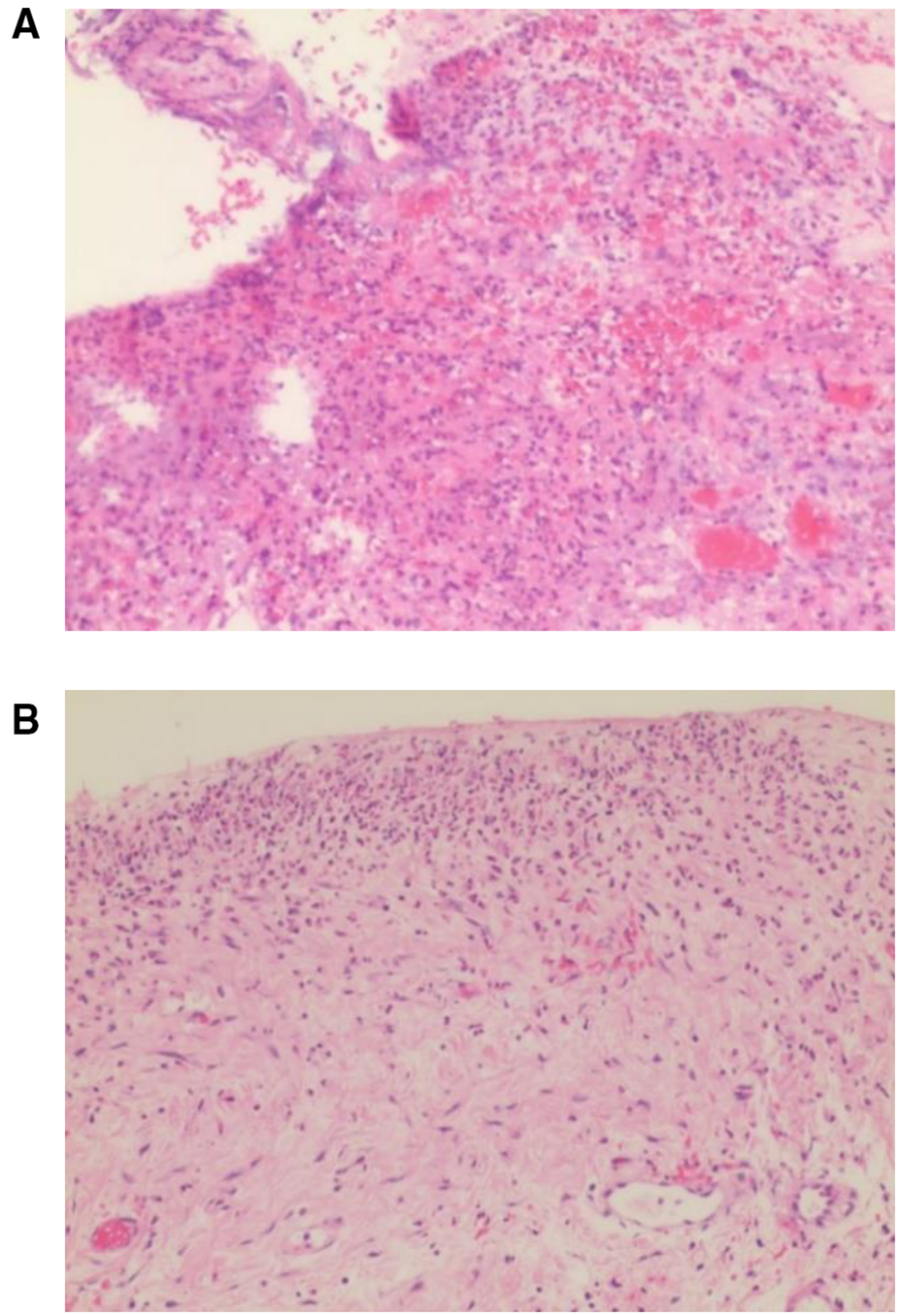
(**A,B**) Pathologic examination showed chronic active inflammatory mucosa of the gastrointestinal tract with erosion and ulcer formation, stromal fibrosis, focal neutrophil, lymphocyte, and plasma cell infiltration. There was no evidence of tumor infiltration in the resected specimen.

The pathological examination showed chronic active inflammatory mucosa of the gastrointestinal tract with erosion and ulcer formation, stromal fibrosis, focal neutrophils, lymphocytes, and plasma cell infiltration. There was no evidence of tumor infiltration in the resected specimens (Figures 8A,B).

**Figure 9:**
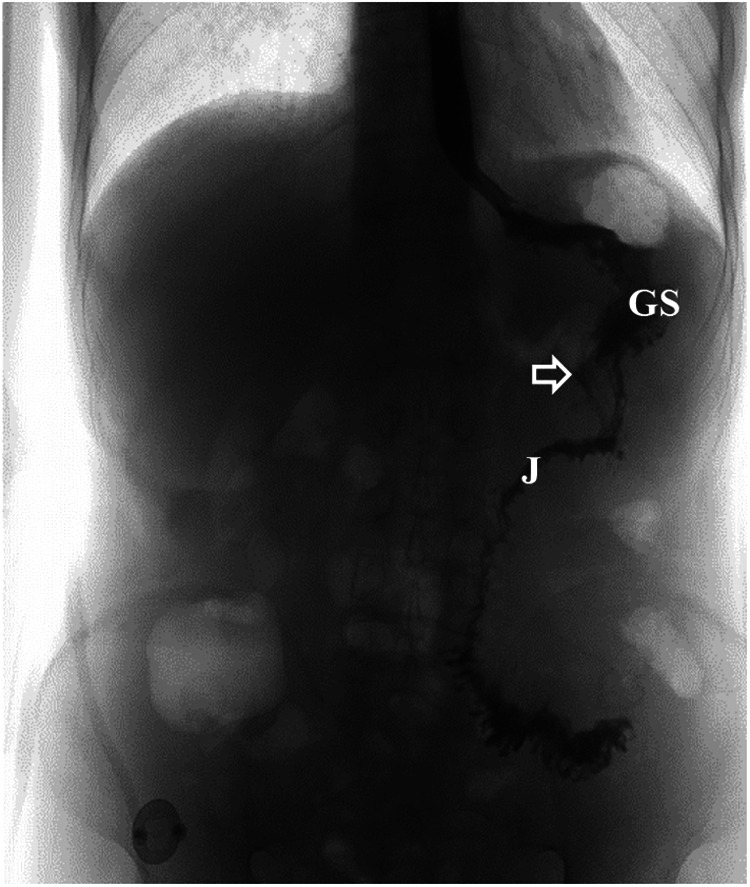
Postoperative imaging findings after surgical treatment of the fistula. Upper gastrointestinal series showed the contrast media entered the gastric stump and passed smoothly through the anastomosis into the jejunum with no obvious fistula (the white arrow indicates the gastrointestinal anastomosis). GS, gastric stump; J, jejunum.

Postoperatively, it was necessary for the patient to fast. Anti-infection treatment, parenteral nutrition, albumin infusion, somatostatin, and gastric mucosal protective agents were administered. The patient was discharged from the hospital 9 days after surgery. An oral contrast study performed 3 weeks later revealed that the contrast media entered the gastric stump and passed smoothly through the anastomosis and into the jejunum with no obvious fistula (Figure 9). At the follow-up examination 4 months after discharge, the patient's abdominal pain and diarrhea had disappeared. He tolerated a normal diet and experienced weight gain of 11 kg.

## Discussion

Duodenal ulcer is the most common cause of benign duodenocolic fistulas (1, 6). The first fully documented benign case was reported by Sanderson in 1,863 (6). Other causes include Crohn's disease (7, 8), ulcerative colitis, ingested magnets or nails (9), duodenal diverticula (3), colonic diverticulum (3), cholelithiasis (10), intestinal tuberculosis (11), pancreatitis, ruptured appendicitis, and regional enteritis (12). Some studies have documented benign duodenocolic fistulas as a complication after the treatment of diseases such as a stomal ulcer after subtotal gastrectomy (13) and migrated biliary stents (14). Our patient had a history of chronic duodenal ulcers and Helicobacter pylori infections. The postoperative pathological examination revealed ulcer formation without tumor cells. Therefore, this case was diagnosed as a duodenocolic fistula because of chronic duodenal ulcer penetration.

The characteristic clinical features of a duodenocolic fistula include diarrhea, abdominal pain, and weight loss (3). Our patient experienced all these symptoms. Diarrhea is the most prominent symptom, and it has been attributed to all of the following causes: colonic contents being regurgitated into the small intestine, thus causing feces to contaminate the intestine and cause bacterial enteritis; the colon contains hydrochloric acid and unconjugated bile acids that irritate the colonic mucosa and cause excessive peristalsis; and reduced transit time or shunting of the contents of the small intestine to the colon (2, 15). Our patient had undigested food in the stool, which is evidence of the contents of the small intestine entering the colon directly without digestion and absorption in the jejunum and ileum (4), thus further confirming the diagnosis of the duodenocolic fistula. Subsequently, his frequent diarrhea led to weight loss. Other clinical manifestations include vomiting, electrolyte imbalances, hypoproteinemia, short bowel syndrome, megaloblastic anemia (16), steatorrhea (17), and osteomalacia (7). Fecal vomiting, which may be related to the pylorus preventing the retrograde flow of colonic contents into the stomach, rarely occurs (5). In 1998, Balaji et al. described a case of rectal bleeding with a fistula eroding the colic vessels in the mesocolon (18). The patient in our case had hematemesis, which was probably caused by peptic ulcer hemorrhaging. Patients with malignant fistulas may have a palpable abdominal mass, whereas patients with benign fistulas have normal physical examination results (4). Clinical symptoms of the primary disease should not be overlooked, and the symptoms that result from the primary disease itself and the fistulous tract should be distinguished.

The most reliable diagnostic tool is a barium enema, which has a sensitivity of 85% to 95% (19). Barium enemas can demonstrate fistulous tracts. Imaging features include retrograde flow of the barium in the hepatic flexure and sudden filling of the small bowel (20). According to the literature, malignant fistulas are characterized by wide linear fistulas and a lack of proximity to the intestinal loops involved in the fistula. These characteristics may be useful for distinguishing between benign and malignant fistulas (21). Additionally, a barium meal can be performed to show the fistula, but it is less sensitive (approximately 40%) than the barium enema. Lower pressure in the small intestine compared to that in the colon may be the reason for the superiority of the barium enema compared to the barium meal (10). Nevertheless, duodenal malformations or a delay in the flow of barium through the duodenum may be visible after barium ingestion. Gastroduodenoscopy and colonoscopy not only allow direct visualization of the fistula for diagnosis but also help to determine the diagnosis of the primary disease. Computed tomography can be used to identify the primary disease at the fistula, identify the origin of the tumor, and distinguish benign from malignant diseases (19). In this case, the fistula was initially identified by gastroduodenoscopy. Furthermore, computed tomography showed no clear boundary between the descending duodenum and the transverse colon and no obvious mass. A subsequent upper gastrointestinal study further confirmed the presence of the fistula.

The fistula is a chronic disease, and the goal of treatment is permanent closure. Various treatments have been used to treat fistulas caused by benign diseases. We collected 15 articles involving 22 cases (Table 1). Operative treatment was performed for 18 of those cases. The treatment of 15 cases involved closure of the fistula. For three cases, right hemicolectomy and ileocolic anastomosis were performed. Three cases were treated with partial gastrectomy and gastrojejunostomy. Although surgery is the main treatment modality, only a few cases of successful nonsurgical treatment have been reported. One case secondary to duodenal ulcer spontaneously recovered, one case originating from Crohn's disease successfully healed after treatment with tacrolimus, and two cases were closed with a through-the-scope clip *via* endoscopy (Table 1). Therefore, it appears that nonsurgical treatment *via* endoscopy is a potentially safer and less invasive approach. To sum up, fistula closure has been the most commonly performed procedure for the treatment of fistulas. Partial gastrectomy combined with gastrojejunostomy can be beneficial for severe adhesions and when dissection of the fistula is difficult (13, 23). Right hemicolectomy can be considered for patients with obstructive lesions in the colon or inflammatory tissues involving the cecum and ascending colon (7, 23). A through-the-scope clip can be attempted for acute perforations with small fistulas when patients have no signs of peritonitis (14, 27). Here, this is a chronic case with big fistula that was not suitable for endoscopic clamping. The intraoperative findings indicated the formation of extensive adhesions and scar tissue. It is hard to employ simple removing fistula. The patient accordingly underwent the resection of the segmental duodenum with distal stomach and partial transverse colon. The Roux-en-Y gastric bypass and the end-to-end intestinal anastomosis were chosen for the reconstruction of the stomach and colon, respectively (Figures 7B,C).

**Table 1:** Details of reported cases of duodenocolic fistula.

No	Years	Age	Gender	Etiology	Site of Fistula		Treatment	Reference	Comments
					Part of duodenum	Colon			
1	1950	52	F	intestinal tuberculosis	Third	Ascending	Closure of fistula	Ogilvie (11)	
2	1950	54	M	intestinal tuberculosis	Third	Hepatic flexure	Closure of fistula	Ogilvie (11)	
3	1960	51	F	Unknow	Second and third	Hepatic flexure	Closure of fistula	Chandler and Longmore (22)	
4	1960	58	M	duodenal ulcer	Second	Not mention	Spontaneous cure	Chandler and Longmore (22)	
5	1960	40	M	duodenal ulcer	Second	Transverse	Polya gastrectomy and closure of fistula	Grayson and O’Connell (23)	^a^
6	1960	33	M	Regional ileitis	Not mention	Hepatic flexure	Right hemicolectomy and closure of fistula	Grayson and O’Connell (23)	^b^
7	1963	76	F	Gall-bladder disease	First	Hepatic flexure	Cholecystectomy and closure of fistula	Dowse (10)	
8	1968	65	F	Unknow	Second and third	Ascending	Closure of fistula	Cuddigan and Edgar (24)	
9	1972	48	F	Spontaneous fistula	Third	Transverse	Closure of fistula	Torrance and Jones (16)	
10	1972	51	M	Spontaneous fistula	Third	Transverse	Closure of fistula	Torrance and Jones (16)	
11	1972	47	F	Spontaneous fistula	Not mention	Not mention	Closure of fistula	Torrance and Jones (16)	
12	1985	80	F	Colonic diverticulum	Second	Hepatic flexure	Closure of fistula	Ferguson and Moncure (3)	
13	1985	76	F	Colonic diverticulum	Second and third	Hepatic flexure	Closure of fistula	Ferguson and Moncure (3)	
14	1985	48	M	Duodenal diverticulum	Second and third	Hepatic flexure	Closure of fistula	Ferguson and Moncure (3)	
15	2004	35	F	Crohn's disease	Second	Transverse	Tacrolimus for the treatment of fistulas	Fukuda et al. (8)	^c^
16	2007	23	F	Crohn's Disease	Second	Not mention	Laparoscopic right hemicolectomy and closure of fistula	El et al. (7)	^d^
17	2013	51	M	Stomal ulcer	Not mention	Transverse	Resection of the distal gastric remnant with the proximal duodenal bulb and a part of the transverse colon, including the fistula	Sawaguchi et al. (13)	^e^
18	2014	44	M	Duodenal ulcer	First	Transverse	Right hemicolectomy, distal gastrectomy surgery with gastrojejunostomy and ileocolic anastomosis, and fistula excision	Kamani et al. (25)	
19	2015	60	M	Duodenal ulcer	Second	Transverse	Closure of fistula	Soheili et al. (26)	
20	2016	72	M	Migrated biliary stent	Second	Not mention	Closed with a through-the-scope clip	Sanchez et al. (14)	
21	2020	3	M	Nail ingestion	Second	Ascending	Closure of fistula	Kassegne et al. (9)	
22	2021	61	F	Unknow	Second	Hepatic flexure	Closed by using an over-the-scope clip	Cakmak (27)	^f^
23	2022	53	M	duodenal ulcer	First and second	Transverse	Resection of the segmental duodenum with distal stomach and partial transverse colon	Own case	^g^

M, male; F, female.

^a^ The hepatic flexure of the colon was bound to the duodenum by many adhesions.

^b^ Obstructive lesion in the proximal transverse colon.

^c^ The patient refused surgery and anti-TNF-α antibody treatment was not effective.

^d^ Colonoscopy showed a stenotic and erythematous area in the proximal transverse colon.

^e^ The patient with fistula developing after distal gastrectomy with B-I reconstruction. The intraoperative findings indicated the presence of severe adhesions between the proximal duodenum and transverse colon. The stomach was reconstructed using the Roux-en-Y method, and the transverse colon was anastomosed in a functional end-to-end manner.

^f^ The fistula mouth was about 8 mm wide.

^g^ The Roux-en-Y gastric bypass and the end-to-end intestinal anastomosis were chosen for the reconstruction of the stomach and colon, respectively.

In conclusion, benign duodenocolic fistulas are rare and are associated with abdominal pain, diarrhea, and weight loss. The most reliable test for determining the presence of a duodenocolic fistula is a barium enema. Benign duodenocolic fistulas are usually treated by closure of the fistula. If obstructions or severe adhesions are present, then right hemicolectomy or partial gastrectomy may be treatment options. In some cases, the placement of an through-the-scope clip *via* endoscopy is a potentially safer and less invasive option. A case of benign duodenocolic fistula secondary to duodenum ulcer is presented. Surgery was performed. Postoperatively, his conditions improved dramatically, and he gained 11 kg. In addition, the etiology, clinical features, diagnosis, and treatment are reviewed.

## Data Availability

The original contributions presented in the study are included in the article/Supplementary Material, further inquiries can be directed to the corresponding author/s.
